# Are new implants for patellar fractures reasonable? A biomechanical comparison of intramedullary locking nails versus tension-band osteosynthesis

**DOI:** 10.1007/s00402-026-06277-5

**Published:** 2026-04-06

**Authors:** Nina Gercek, Charlotte Arand, Christian Glockner, Michael Nienhaus, Erol Gercek, Johannes Hopf, Pol Maria Rommens, Dominik Gruszka

**Affiliations:** 1 University Medical Center of the Johannes Gutenberg University Mainz, Mainz, Germany; 2 RheinMain University of Applied Sciences, Wiesbaden, Germany

## Abstract

**Background:**

Patellar fractures are predominantly treated surgically by open reduction and internal fixation using classic tension band osteosynthesis. Biomechanical testing was conducted to examine transverse patellar fracture stabilization using locked intramedullary nail systems in comparison to classic tension band osteosynthesis.

**Methods:**

Twenty-four Sawbones^®^ with transverse patellar fractures type AO 34-C1.1 were divided equally into three test groups. Three principles for fracture stabilization were used: classical tension band osteosynthesis and newly developed locked intramedullary double nail- and single nail- prototypes. Knee motion (0°/0°/90°) was simulated in a dynamic test set-up using a “single muscle model” by applying tensile forces up to 300 N. The widening and the symmetry of the fracture gap were investigated over 1000 motion cycles using a video-optical system. The significance level was set at α = 0.05 for all statistical evaluations using the Bonferroni–Holm corrected unpaired unilateral t-test and mixed ANOVA with post-hoc Tukey test.

**Results:**

The fixation principles showed different maximum fragment displacements of M = 2.04 ± 0.67 mm using classical tension band osteosynthesis, M = 1.34 ± 0.74 mm using intramedullary single nail system and M = 0.55 ± 0.31 mm using intramedullary double nail system. There was a statistically significant change in width of the fracture gap in flexed knee position comparing tension band osteosynthesis and double nail system (*p* = 0.0015) and comparing single nail and double nail system (*p* = 0.02). Furthermore, the tension-band osteosynthesis showed a significantly greater divergence from symmetry in the fracture gap compared to both intramedullary treatment methods (*p* < 0.001).

**Conclusions:**

Prototypes of locked intramedullary nail systems showed comparable fixation results for transverse patellar fractures compared to tension band wiring. They showed more symmetrical fracture gap openings during biomechanical testing and offered significantly higher stability to the fragments.

## Introduction

Patellar fractures are considered rare with an incidence of 14/100,000 inhabitants in Germany. Gold standard and most applied method is a surgical treatment using classical tension-band wiring osteosynthesis (TBW) and Kirschner wires [1–5]. Recently, there has been a rising interest in studying surgical treatment alternatives [6].

Another accepted fixation method for transverse patellar fractures is the combination of two cannulated screws combined with tension band wiring [7–9]. However, biomechanical studies show technical complications, such as implant loosening, migration or failure with secondary fracture displacement [10–12]. In the clinical setting, such complications also lead to postoperative pain and soft tissue irritation. These complications enforce reoperation and usually necessitate removal of the implant [13–15]. Plating with specific angle-stable plate systems have been introduced into the orthopedic market [16–18]. Plating provides better biomechanical and clinical outcomes compared to tension band wiring [19–23], but extensive soft tissue preparation is necessary to attach the angle-stable plates [4]. To date, plating of patellar fractures is not widely used, and long-term results are lacking.

Intramedullary nail systems are considered to reduce the amount of soft tissue preparation and address more central transmission of force vectors [24]. Friedl et al. already introduced an intramedullary patellar XS-nail and showed in their initial clinical study that out of 49 patients, 69% achieved excellent results after nailing [25]. The present study aims to demonstrate comparable biomechanical properties using internal fixation for transverse patellar fractures with a novel intramedullary nail implant system.

## Materials and methods

### Bones

Twenty-four left sided composite patellar models of 4th generation (Sawbones, Vashon, Washington, USA) were used for biomechanical testing. Vise tabs were manufacturer-specific integrated at the upper and lower pole of the patella for experimental setups.

Using a purposely designed template all patellae were osteotomized to create a fracture type AO/OTA 34 C1.1. The samples were numbered and randomly assigned into three groups using Microsoft^®^ Excel’s random distribution function, treated with the following principles:

- Group 1: double-nail-system (2N).
- Group 2: single-nail-system (1N).
- Group 3: Classical tension-band wiring (C).

**Fig. 1:**
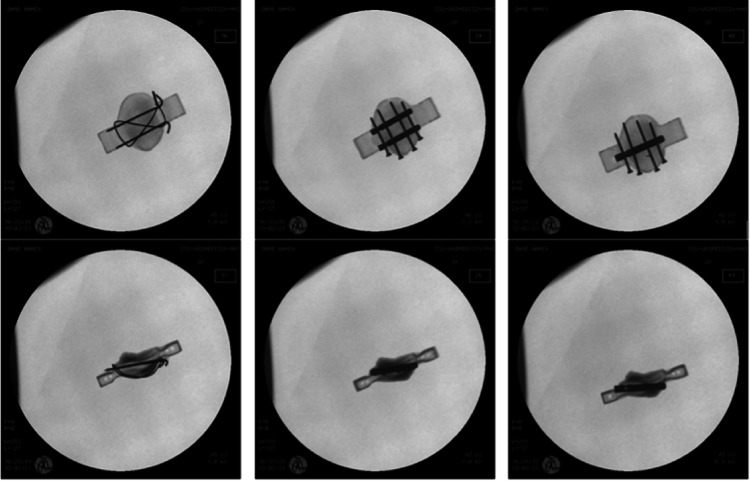
Conventional radiological image of the patella specimens (a.p. and lateral view), left: classical tension-band wiring (C), middle: double-nail-system (2N), right: single-nail-system (1N)

After internal fixation each patella was controlled radiologically as seen in Fig. 1.

### Implants

Initial prototypes of the intramedullary nail system were developed in collaboration with the RheinMain University of Applied Sciences (Wiesbaden, Germany) and tested in the biomechanics laboratory at the University Medical Center of Johannes-Gutenberg University (Mainz, Germany).

The novel intramedullary nail implants were additively manufactured (3D printing) from stainless steel in cooperation with Kegelmann Manufacturing GmbH & Co (Rodgau, Germany). The implant for single-nail-system (1N) has a length of 45 mm, a diameter of 6 mm, and four locking holes with a diameter of 2.7 mm each and a long hole at the distal end. The implant for double-nail-system (2N) is slightly shorter (40 mm) and has a smaller diameter (5 mm), with the same locking option as above. Classical tension band wiring was applied using two K-wires (1.8 mm) and formatting a wire cerclage (1.25 mm).

**Fig. 2:**
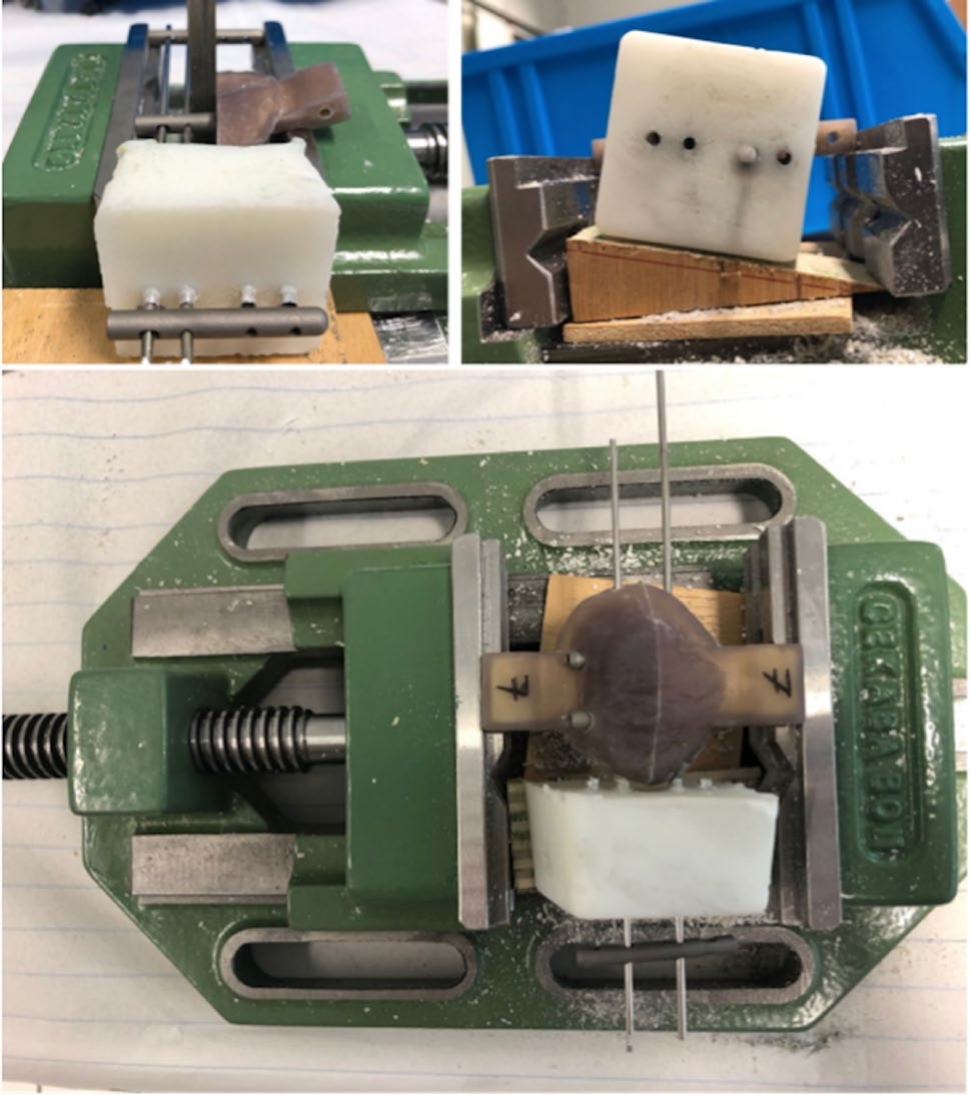
Custom-made aiming device for locking intramedullary nail system

The double-nail-system was designed to create a grid-like structure within the bone, which was expected to lead to higher stability and reduce rotation of fragments. In addition, a custom-made aiming device for implantation was developed to insert and fix the nails into the bone (Fig. 2). Nail locking was performed with 2.7-mm cortical screws (Königsee Implantate GmbH, Allendorf, Germany), placing two screws proximally and two distally.

### Test set-up

**Fig. 3:**
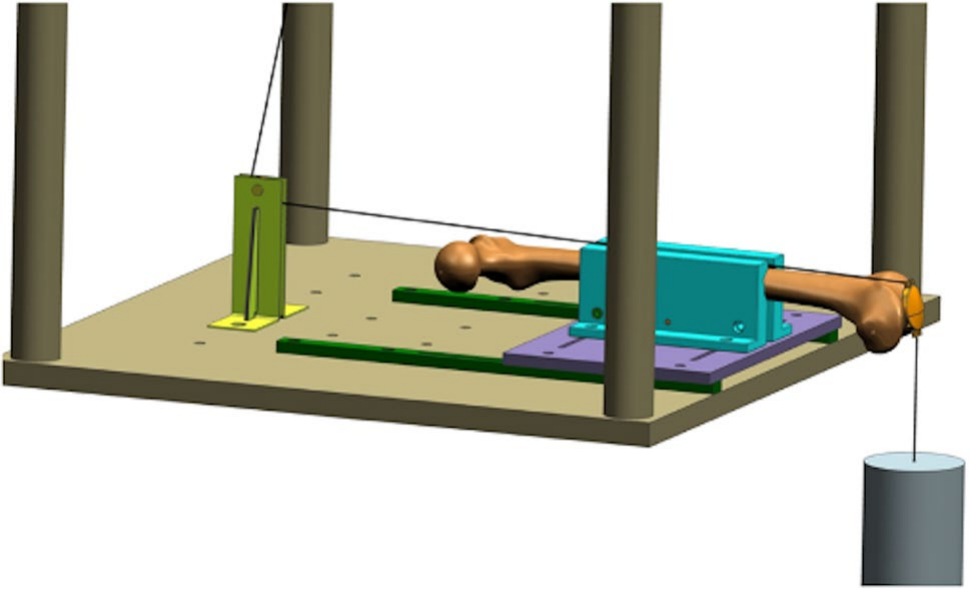
Test set-up (Hochschule-RheinMain, R. Helfrich)

The simulated knee motion using a single-muscle model was based on established test set-ups from Schnabel et al. [26]. Steel cable wires, which simulate the upper leg extensor, were connected to the lifting arm of the testing machine. A weight with a mass of 17.4 kg was suspended on steel cable wires to simulate a lower leg. This construct worked with a pull force of 300 N per cycle [27](Fig. 3) for extension and flexion motion of knee joint 0°−0°−90° against gravity without influence of the entire body weight [10, 26, 28, 29].

**Fig. 4:**
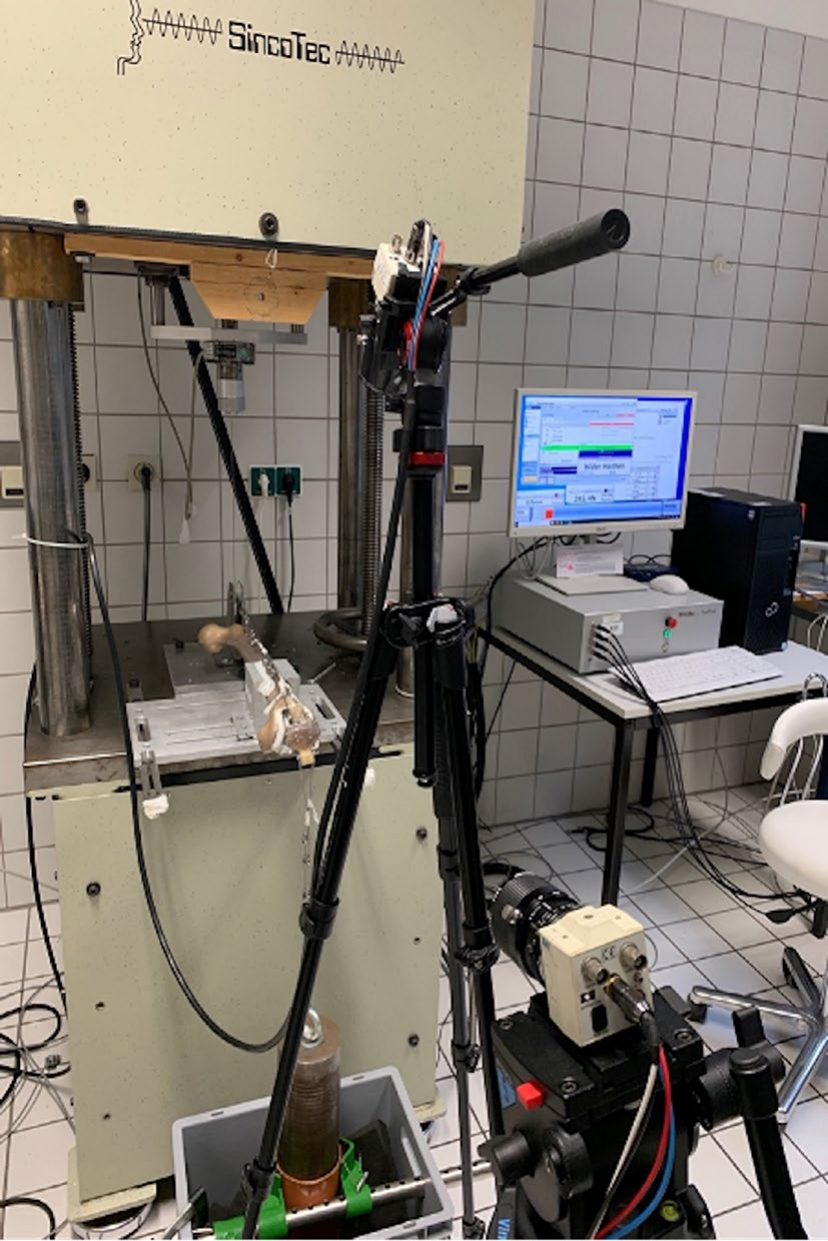
Experimental set-up, SincoTec testing-machine

For the experimental set-up (Fig. 4), the 4th generation femur (Sawbones, Vashon, Washington) was placed and screwed horizontally in its natural orientation with 10° cranially femoral head rotation onto the table of the testing-machine (SincoTec GmbH, Clausthal-Zellerfeld, Germany). A pulley behind the femur worked as a guide and force redirection for the steel cable (thickness 1.2 mm) to simulate the upper leg. At the other end of the steel cable, the patella was attached to its upper pole. The lower patellar pole was attached to the weight via a steel cable (thickness 1.5 mm). A guide rail was positioned to avoid swinging of the hanging weight. To improve sliding between patella and femur, the distal articular surface of the femur was provided with a surface femoral shield.

### Testing protocol

The biomechanical testing of 24 specimens was performed with a servo-hydraulic testing machine using a cyclic loading test. The traction of the quadriceps femoris muscle was considered clinically relevant. A control protocol was programmed with linear cylinder to simulate knee movement of a seated patient between 90°-flexion up to full extension. The patella was pulled into extension position at a speed of 2 mm/s and moved back to starting position at a speed of 3 mm/s, resulting in a test duration of 9.5 h for 1000 motion cycles per sample. The control protocol automatically stopped after each 50 cycles for image documentation until 1000 cycles were reached or failure criterion occurs. Failure was defined to occur when proximal and distal fragments of the fracture showed a dislocation larger than 2 mm during cyclic loading tests [10, 30].

In addition, failure occurred when the osteosynthesis material broke or dislocated and furthermore a peri-implant fracture or a fracture elsewhere on the specimen occurred. An exception was made for other types of material weaknesses, such as rupture of the used steel cables. If this happened, the test stopped to replace materials and started again with newly supplied specimens.

**Fig. 5:**
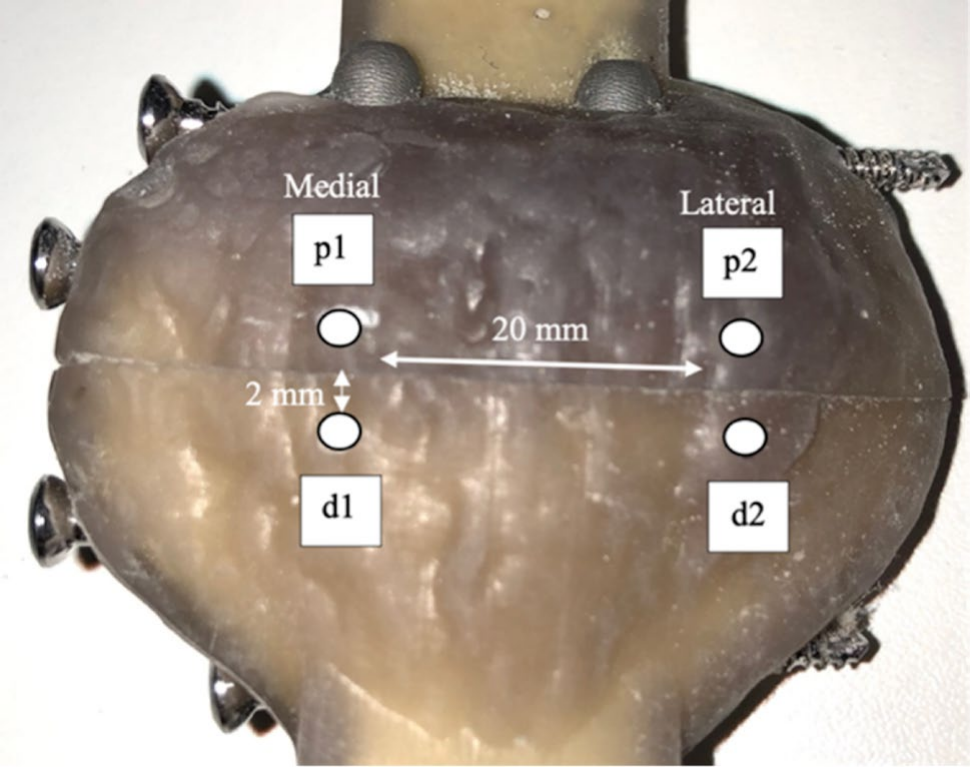
Optical marker on medial and lateral fracture side, *p* proximal, *d* distal

Each patella was marked with four optical markers near the fracture gap (Fig. 5). Two cameras optically tracked motion of the medial and lateral side of the fracture fragments in extension and flexion. The changes in the fracture gap were evaluated using a motion-analysis-program SimiMotion (Simi-Reality-Motion-Systems GmbH, Unterschleißheim, Germany).

### Statistical analysis

Statistical analysis was performed using SPSS software package 27, IBM SPSS Statistics for Macintosh, Version 27.0 (IBM, Armonk, NY, USA). The significance level was set at α = 0.05 for all tests. The primary target value was the change in fracture gap width on the medial and lateral fracture sides after 100, 500 and 1000 cyclic loading tests. Bonferroni–Holm corrected unpaired unilateral t-tests were used to assess the movement in the fracture gap. In further analysis the mixed ANOVA, analysis of variance, was used as an omnibus test to examine differences in the symmetry of the fracture gap with respect to other factors. A post-hoc Tukey test determined differences.

## Results

The mean change (± standard deviation) of medial and lateral fracture gap widths in extension- and flexion-positions showed that the double-nail system has statistically significant lower displacement of the fracture gap widths compared to both the classical tension-band osteosynthesis (*p* < 0.05) and the intramedullary single-nail (*p* < 0.05).

**Table 1:** s hows the results of the Bonferroni–Holm corrected unilateral unpaired t-tests after 1000 motion cycles on the medial and lateral fracture side depending on the knee joint position (Ext/ Flex) comparing each test group

Fracture side	C, Ext	1 *N*, Ext	2 *N*, Ext	corrected *p*-value	C, Flex	1 *N*, Flex	2 *N*, Flex	corrected *p*-value
medial	0.98 (0.59)	0.88 (0.63)		*0.38*	0.96 (0.37)	1.23 (0.60)		*0.85*
	0.98 (0.59)		0.28 (0.18)	0.009**	0.96 (0.37)		0.46 (0.29)	0.01*
		0.88 (0.63)	0.28 (0.18)	0.03*		1.23 (0.60)	0.46 (0.29)	0.009**
lateral	1.76 (0.80)	0.87 (0.66)		0.029*	2.04 (0.67)	1.34 (0.74)		0.032*
	1.76 (0.80)		0.33 (0.22)	0.0015**	2.04 (0.67)		0.55 (0.31)	0.0015**
		0.87 (0.66)	0.33 (0.22)	0.03*		1.34 (0.74)	0.55 (0.31)	0.02*

Unpaired unilateral T-test with Bonferroni–Holm corrected p-values in extension- and flexion-position (Ext/ Flex) of the three groups (C, 1N, 2N), mean values (standard deviation, SD) of the gap widths in mm of the medial and lateral fracture sides after 1000 motion cycles

Significant values are in bold (*p < 0.05, **p < 0.01, ***p < 0.001)

In the flexion position, mean gap widths (M) differed significantly between the two constructs. After 1,000 cycles, the double-nail system demonstrated a mean medial fracture-side gap width that was 0.50 mm smaller than that of classic tension-band osteosynthesis (95% CI [0.14; 0.86], *p* = 0.01).

Similar results were observed on the lateral fracture side, where the mean gap width of tension-band osteosynthesis was 1.49 mm larger than that of the double-nail system (95% CI [0.98; 1.98], *p* = 0.0015), as shown in Table 1.

When comparing both intramedullary treatment methods, a statistically significant difference in the change of gap width in the flexion position was observed on both fracture sides. On the medial side, the single-nail system showed a mean gap width that was 0.77 mm larger than that of the double-nail system (95% CI [0.26; 1.23], *p* = 0.009). On the lateral side, the gap width of the single-nail system was on average 0.79 mm larger (95% CI [0.16; 1.43], *p* = 0.02).

Considering the change of width on the medial fracture side in extension and flexion position of the knee joint, the single-nail system could not be significantly distinguished from tension-band osteosynthesis (*p* > 0,05). The assumption was not confirmed that the single-nail-system showed lower mean values of the fracture gap width than classic tension-band. The gap width of the single-nail-method on the medial fracture side was on average 0.27 mm larger than that of the tension-band in flexion position (95% CI [−0.81; 0.27], *p* = 0.85).

On the lateral fracture side in extension- and flexion-position, there were statistically significant differences in fracture gap width between both test groups (*p* < 0.05). The mean values of the gap widths in 90°-flexion of classical tension-band were on average 0.7 mm larger compared to those of the single-nail-method (95% CI [−0.05; 1.46]), *p* = 0.032).

**Fig. 6:**
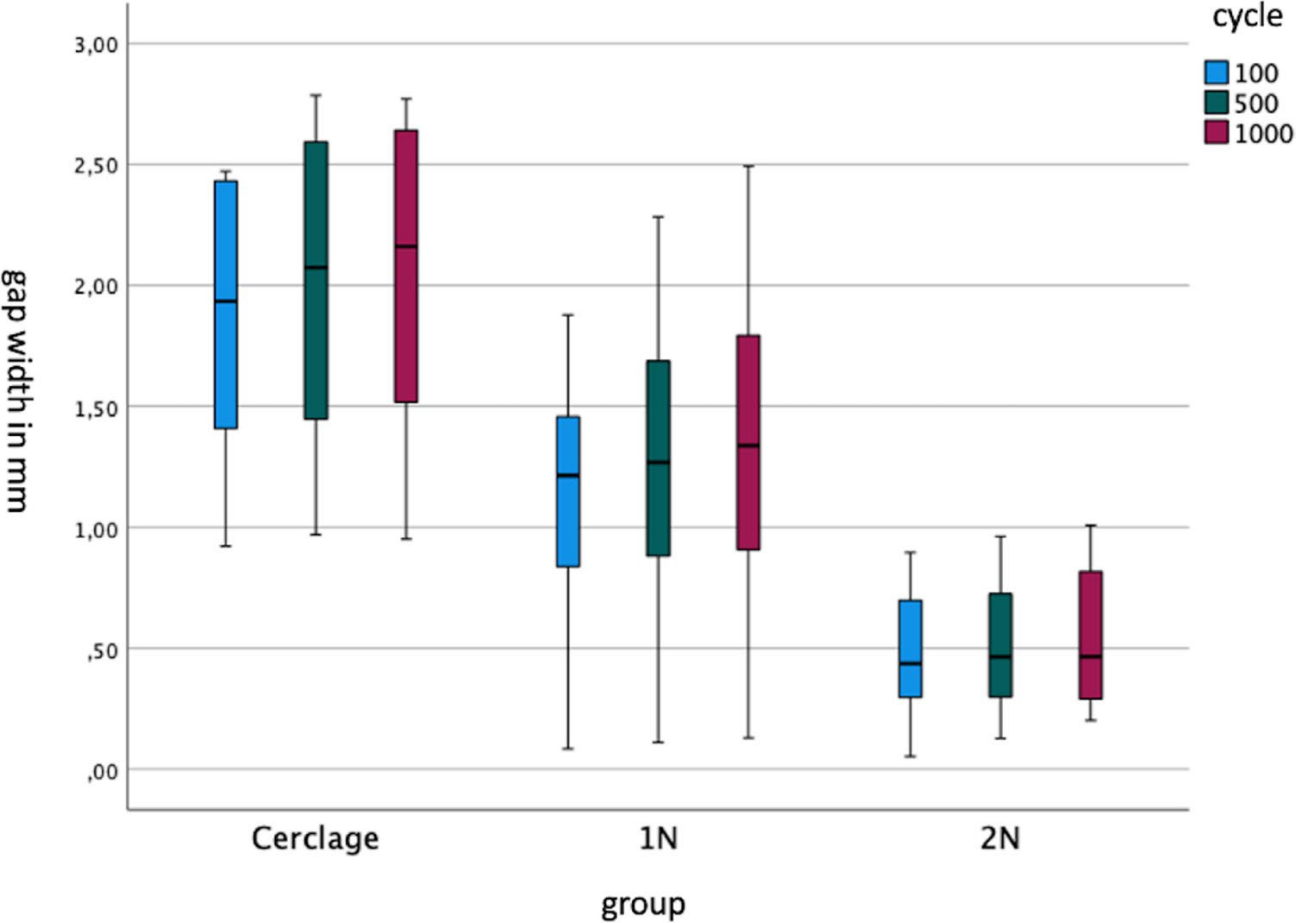
Boxplot diagram of the gap widths in mm of the lateral fracture side of the three test groups: Cerclage, 1N, 2N after 100, 500 and 1000 motion cycles in flexion position of the knee joint

Furthermore, implant failure of tension-band osteosynthesis was demonstrated on the lateral fracture side with a fracture gap larger than 2 mm, whereas no failure occurred with the intramedullary methods (Fig. 6).

**Fig. 7:**
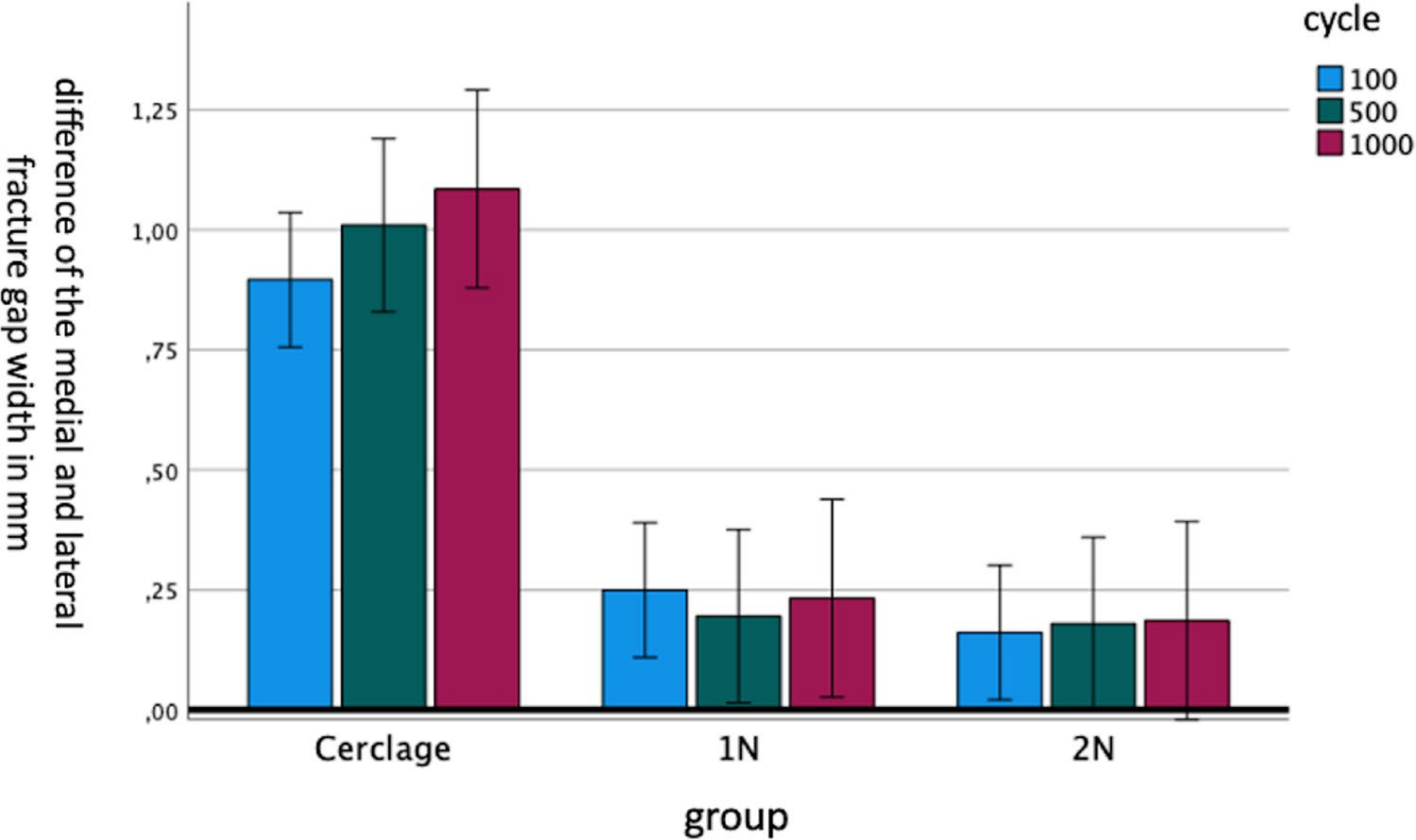
Difference in symmetry of the fracture gap after 100, 500 and 1000 motion cycles for the three test groups: Cerclage, 1N, 2N

In summary, the double-nail-system stood out from both other groups and showed a statistically significant smaller change in gap width after biomechanical testing. The evaluation of the symmetry of the fracture gap was represented by the differences between lateral and medial fracture gap width. A large difference meant that the gap is not symmetrical. This was evaluated with a mixed ANOVA and post-hoc tukey test (Table 2). Relating to the symmetry of the fracture gap measured by the tukey test, the tension-band osteosynthesis showed a statistically significant greater divergence of symmetry than both intramedullary nail systems (*p* < 0.001). Between both intramedullary systems there was no statistically significant difference relating to the symmetry (*p* = 0.897). This difference is graphically illustrated in Fig. 7.

**Table 2:** The results of post-hoc Tukey test

Group (I)	Group (J)	mean difference (I-J)	Standard errors	Significance	95% confidence interval Lower limit	95% confidence interval Upper limit
Cerclage	1N	0.771	0.113	< 0.001	0.487	1.056
Cerclage	2N	0.822	0.113	< 0.001	0,537	1.106
1N	2N	0.050	0.113	0.897	−0.234	0.335

## Discussion

Currently, various surgical options are available for operative treatment of transverse patellar fractures. Classical tension-band osteosynthesis as the gold standard method is associated with high complication rates and plate fixation needs extensive soft tissue preparation. Intramedullary fixation methods have proven to be less invasive and due to implant positioning closer to the force vectors, to provide a higher stability. Therefore, a biomechanical testing of intramedullary nail-systems on synthetic bones is performed to demonstrate comparable stability compared to classical tension-band osteosynthesis.

The aim of operative stabilization of patellar fractures is allowing early functional loading [31]. Many studies have already dealt with maximum loads on the knee joint to evaluate the forces that can lead to fractures of the bones or implants (“load to failure”) [10, 32–34]. However, clinical postoperative mobilization should be considered, which is carried out with a high number of cyclic loads. Previous studies examined the mass of the test weight and the tension that acts on the quadriceps tendon to extend the knee from 90° flexion. Here, Hungerford et al. was able to show with a theoretical analysis that 28 kg were necessary to extend the knee [35]. Kaufer et al. experimentally showed that 17–26 kg were necessary for full knee extension [36]. According to Scilaris et al. and Nienhaus et al. force peaks between 300 and 400 N occur during cyclic loading simulating postoperative mobilization [28, 37]. Carpenter et al. found that a weight of 17–30 kg was sufficient to move the knee joint from 90° flexion to full extension against gravity [10]. In this study, a uniform cyclic load over 1000 movement cycles with a test weight of 17.4 kg and forces up to 300 N is simulated.

Friedl et al. describes the principle of interfragmentary compression using the XS tension-band nail, which has similarities to the intramedullary nails used in this study [24, 25, 38, 39]. First XS-nails already have been introduced into the orthopedic market. The authors were able to show superiority of the tension-band compression nail over classical tension-band osteosynthesis and postulated that intramedullary systems bring a more even distribution of pressure to the fracture components. This can be endorsed with the present study which shows a significantly more symmetrical fracture gap using an intramedullary nail system compared to the gold standard. Clinical trials showed that treatment with the XS nail achieved more than 65% excellent results. However, there was no significant difference regarding to the dehiscence of fracture parts using one or two intramedullary nail implants [25]. This can be disproved by the data from the present study, which shows a statistically significant difference regarding to the mean fracture gap widths, whereas the double nail system shows smaller fracture gaps after cyclic loading (*p* < 0.05). Further studies are needed to examine the influence on stability either of the number of implants as well as the number of locking bolts or the diameter of the implant.

Biomechanical studies are conducted under prevailing laboratory conditions. The osteotomy to simulate the transverse patellar fracture removes material from the proximal and distal fragments. Furthermore, the fracture surface by osteotomy is flat and smooth. There is a loss of bone stability, but not a loss of bone material when fracturing a human bone. However, studies on osteosynthesis procedures for transverse patellar fractures with human cadavers and artificial bones also perform osteotomies to simulate fractures [33, 37, 40].

The results of the present study suggest that intramedullary fixation methods in the treatment of transverse patellar fractures are superior to the current gold standard of classical tension-band osteosynthesis. The present results are consistent with the findings of Nienhaus et al., who found no significant differences of the change in width of the anterior fracture gap between a similar intramedullary single nail prototype and the TBW method in combination with cannulated screws based on cadaver testing [37]. However, they were able to show that after a load of 2500–5000 cycles, the gap on the articulating fracture side was significantly smaller (*p* < 0.05) with the nail method, supporting the hypothesis that an intramedullary model is biomechanically superior to tension-band techniques.

In addition, tension-band osteosynthesis showed a fracture gap of more than 2 mm on the lateral fracture side (Fig. 7) which is reported with higher postoperative risk of poor clinical outcome and increasing risk of osteoarthritis [10, 30].

Further studies according to the symmetry of the fracture gap are missing. It must be evaluated if the amount of asymmetry has an outcome on the clinical result. The present study shows a superiority of the intramedullary systems with a statistically more symmetrical fracture gap.

A strength of the present study is that the developed implantation procedures were largely standardized across all three test groups. Furthermore, the costs of using artificial bones are lower compared to studies using human specimens due to lower costs of the specimens and lower environmental costs due to lower energy demand for preservation of specimens for a long period of time according to a good research practice. The collected data does not show any strong outliers or irregularities. The results demonstrate a significant trend in evaluating the stability of the treatment methods and serve as guidance for further biomechanical and clinical studies.

### Limitations

This study has several limitations that must be considered when interpreting the results. First, synthetic composite bone models (“saw bones”) were used instead of fresh-frozen human cadaver specimens, which remain the biomechanical gold standard for evaluating implant performance. Although saw bones offer high reproducibility and consistent material properties, they cannot fully replicate the complex anisotropy and heterogeneity of human bone, particularly in osteoporotic conditions. Moreover, the small sample size within each test group limits the statistical power to detect subtle differences between implant designs. The experiments were also performed in the absence of soft tissues, thus neglecting the stabilizing and damping effects of surrounding ligaments, tendons, and muscles. Finally, the study design did not account for biological healing or bone remodeling processes, which may critically influence implant fixation and long-term performance in vivo. Furthermore, the influence of individual implant characteristics—such as the number and diameter of intramedullary nails and the configuration of locking screws—was not examined in detail and should be addressed in future studies to better delineate their specific biomechanical contributions. These differences in material composition and the lack of biological adaptation warrant cautious interpretation of the findings and constrain direct clinical transferability.

## Conclusions

The intramedullary double-nail prototypes using cortical screws for interlocking show significantly higher stability and significantly more symmetrical fracture gap under cyclic loading compared to classical tension-band osteosynthesis. The project of intramedullary nailing systems after patellar fractures is still in the early stages of development. Further biomechanical studies should be conducted to assess the biomechanical properties and safety of the proposed method of fixation for patellar fractures in human specimens prior to its commercialization.

## Data Availability

All data supporting the findings of this study are available within the paper.
